# An ecologically-controlled exoskeleton can improve balance recovery after slippage

**DOI:** 10.1038/srep46721

**Published:** 2017-05-11

**Authors:** V. Monaco, P. Tropea, F. Aprigliano, D. Martelli, A. Parri, M. Cortese, R. Molino-Lova, N. Vitiello, S. Micera

**Affiliations:** 1 The BioRobotics Institute, Scuola Superiore Sant’Anna, Pisa, Italy; 2 IRCSS Don Carlo Gnocchi Foundation, Firenze, Italy; 3 Department of Mechanical Engineering, Columbia University, New York, NY 10027 USA; 4 Bertarelli Foundation Chair in Translational NeuroEngineering, Center for Neuroprosthetics and Institute of Bioengineering, School of Engineering, Ecole Polytechnique Federale de Lausanne, Lausanne, Switzerland

## Abstract

The evolution to bipedalism forced humans to develop suitable strategies for dynamically controlling their balance, ensuring stability, and preventing falling. The natural aging process and traumatic events such as lower-limb loss can alter the human ability to control stability significantly increasing the risk of fall and reducing the overall autonomy. Accordingly, there is an urgent need, from both end-users and society, for novel solutions that can counteract the lack of balance, thus preventing falls among older and fragile citizens. In this study, we show a novel ecological approach relying on a wearable robotic device (the Active Pelvis Orthosis, APO) aimed at facilitating balance recovery after unexpected slippages. Specifically, if the APO detects signs of balance loss, then it supplies counteracting torques at the hips to assist balance recovery. Experimental tests conducted on eight elderly persons and two transfemoral amputees revealed that stability against falls improved due to the “assisting when needed” behavior of the APO. Interestingly, our approach required a very limited personalization for each subject, and this makes it promising for real-life applications. Our findings demonstrate the potential of closed-loop controlled wearable robots to assist elderly and disabled subjects and to improve their quality of life.

Balance and postural control are achieved by implementing sophisticated motor control strategies to ensure stability and prevent falling^1^,^2^. The natural aging process alters the ability to address unexpected perturbations of balance, thus increasing the probability of falling^3^,^4^. Age-related diseases and traumatic events can further exacerbate the inability to maintain balance, leading to a dramatic increment of both the risk of balance loss and the severity of related accidents^4^,^5^,^6^. The consequences of falls are costly for patients and for society.

In addition to prevention programs based on training^7^,^8^, and behavior and environmental modifications^9^, the effectiveness of which are generally acknowledged, advanced wearable robotic systems could also reduce the risk of falling. For example, novel approaches are currently under investigation to properly design and control transfemoral prostheses aimed at reducing falls in amputees^10^,^11^. However, these solutions cannot be transferred to all fragile and prone-to-fall subjects. On the other hand, robotic exoskeletons could represent a more general and useful solution for a larger cohort of subjects. Nonetheless, despite their potential benefits, stakeholders still notice that the minimization of fall risk is one of the most important factors not yet addressed in the design of these active orthosis^12^.

One of the main problems of current exoskeletons, limiting their use, is the poor human-machine interaction^13^. This issue is particularly important when – as in our case – the users still retain important voluntary abilities. In fact, prone-to-fall subjects are still able to generate “countermeasures” to falling events but not strong and fast enough to avoid these events in most of the cases. In this situation, it is necessary to develop ecological and symbiotic solutions able to help only when necessary (onset of imminent fall) providing only an extra “delta” of response (not substituting by “assisting when needed” the subjects). The hybrid subject-exoskeleton must become a “symbiotic” union with the artificial system providing benefits with no (or very limited) disturbance to the subjects.

In this study, we show that this approach can be implemented improving balance recovery in elderly people and transfemoral amputees. In particular, these two groups of subjects represent persons whose risks of fall ranges from moderate to severe^3^,^4^,^14^,^15^, thus they can be considered as potential users who can take advantages from a wearable assistive device. To achieve this goal a novel control algorithm^16^, leading an Active Pelvis Orthosis^17^ (APO; Fig. 1 and Fig. S1, Supplementary Materials and Methods, Section B), was designed to: i) be transparent to the user’s intended movements during unperturbed motor tasks, ii) identify in real time the onset of an unexpected slipping-like perturbation^16^ (Fig. 2A) and iii) generate countermeasures to restore user’s stability against falling.

## Results

The proposed assistive strategy was tested with eight elderly persons (68.9 ± 5.1 years old) and two transfemoral amputees (65.5 ± 6.4 years old). They were asked to manage unexpected slipping-like perturbations (i.e., one of the most common causes of falling^15^) delivered while walking at their preferred speeds (elders: 0.89 ± 0.11 m/s; amputees: 0.69 ± 0.06 m/s; see also Table S1). The mechanical perturbations were delivered by a mechatronic platform designed to expose enrolled participants to well-controlled experimental conditions (see also Materials and Methods, Section A and Supplementary Materials and Methods, Section A)^18^.

**Figure 1:**
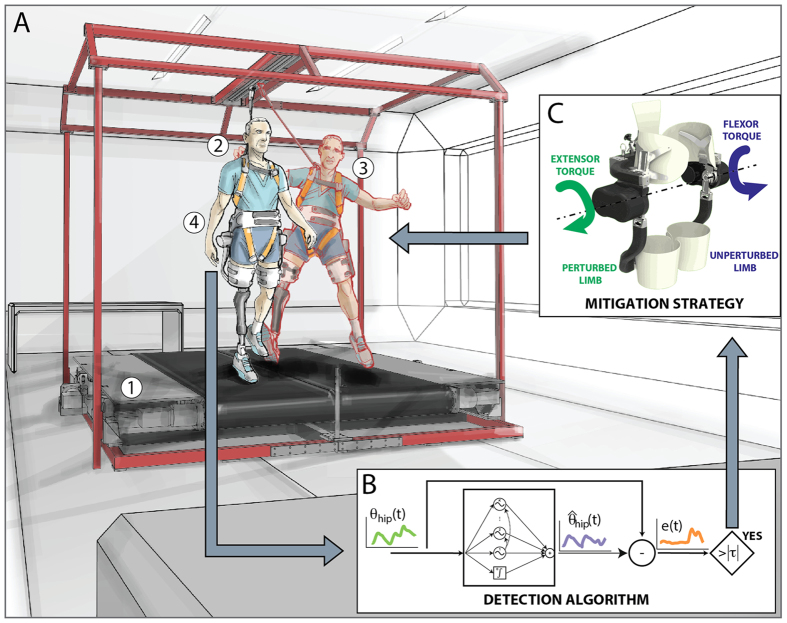
Control strategy. (**A**) The mechatronic platform (1) provides sudden and unexpected slipping-like perturbations while the subject was steadily walking (2). The subject’s balance control was hence challenged (3) and, accordingly, the Active Pelvis Orthosis (4) could supply the assistive strategy for stability recovery. (**B**) The balance loss is detected in real time by an algorithm running in the APO control unit and comparing the actual hip angles of the robot (i.e., θ) with those predicted by a pool of adaptive oscillators (i.e., 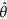). (**C**) Once the slipping-like perturbation was detected, counteracting torques were supplied by the APO at the hip joints to promote balance recovery. Panel C was created by Arch. Alessio Tommasetti Panel C was created by Mr. Francesco Giovacchini.

During the experimental sessions, subjects managed unexpected slippage wearing or without wearing the APO. In the former case, the exoskeleton was used either in zero-torque modality (Z-mode trials), that is, no assistance was provided to the users, or to supply assisting torques at hip joints after detecting postural transitions (A-mode trials). The A-mode consisted in applying synchronous extensor and flexor torques (Fig. 1C) based on the subjects’ body weight, to the perturbed (PL) and the unperturbed (UL) limbs, respectively, in order to counteract the downward displacement of the center of mass (COM; Materials and Methods, Section B; as representative examples, see Movie S1 and Movie S2). In the latter case, to test the unobtrusiveness of the APO during the balance recovery, a subset of the enrolled elderly subjects (n = 5) was asked to manage slipping-like perturbations without wearing the exoskeleton (no-APO trials), as well.

### Analysis of the biomechanical response during balance recovery

When a perturbation was applied, both elderly subjects and amputees showed a significant change of their lower limb kinematics. In particular, the hip and the ankle of the PL were more flexed compared to the steady locomotion (Fig. 3A and B).

**Figure 2:**
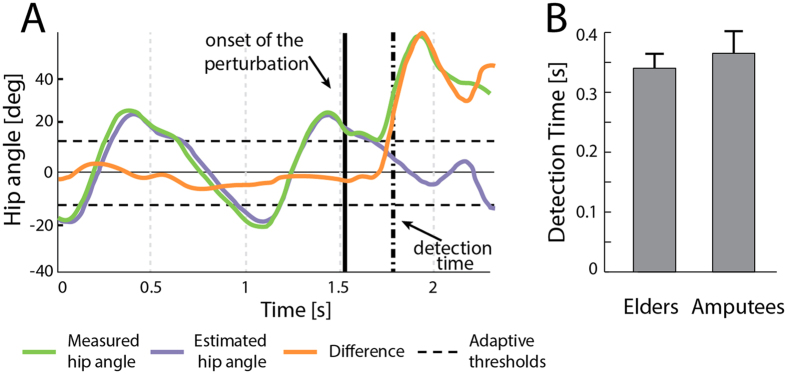
Real time detection of slipping-like perturbations. (**A**) The adaptive-threshold based algorithm analyses the difference between measured (light green) and estimated (purple) hip joint angles. If the error signal (orange) is over the thresholds (dashed lines), a balance loss is detected. (**B**) Detection time obtained during the experimental trials for the elderly subjects and amputees (mean values ± SD).

When the APO was set in A-mode the early modifications of the leg kinematics allowed the detection algorithm to identify balance loss in about 350 ms (Fig. 2B) and to enable the mitigation strategy. This assistive strategy generally ended during the swing phase of the PL (~650 ms).

**Figure 3:**
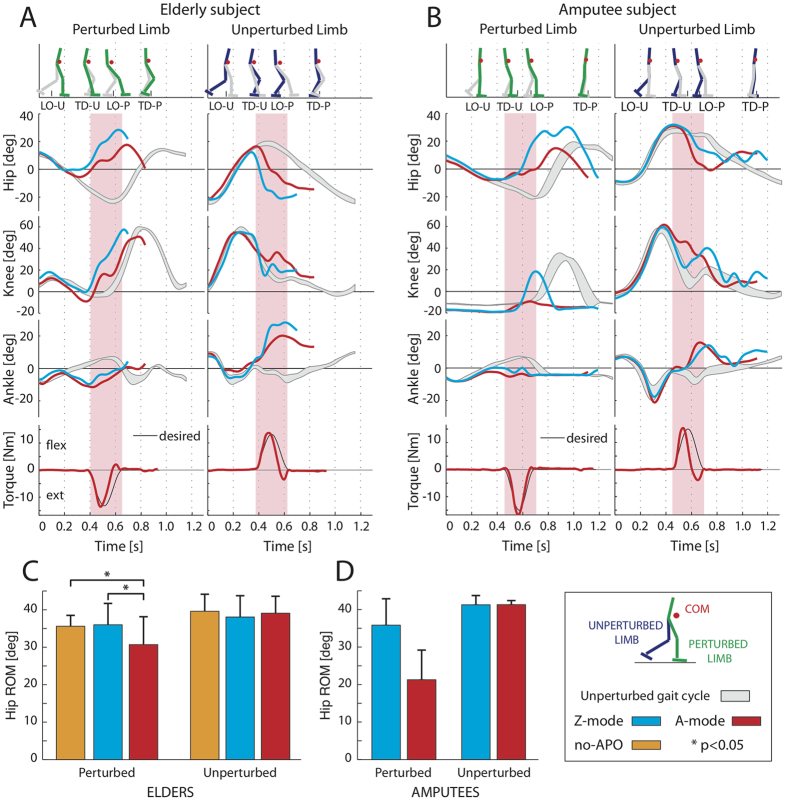
Kinematic patterns at leg joints. (**A** and **B**) Joint angles (hip, knee and ankle) at PL and UL are depicted for three experimental conditions, for one elderly subject and one amputee: i) steady locomotion (mean values ± SD, grey bands); ii) Z-mode (cyan lines); iii) A-mode (red lines). Stick diagrams (on the top; PL and UL are indicated in green and blue, respectively) and APO torques (on the bottom) are shown for the A-mode condition. Pink vertical bands represent the time-intervals corresponding to the enabled assistive torques. (**C**) The perturbed and unperturbed hip ranges of motion during the no-APO, Z- and A-modes trials (orange, cyan and red bars, respectively) are shown for elderly subjects (mean values ± SD). The label * indicates a significant (p < 0.05) difference among trials. (**D**) The perturbed and unperturbed hip ranges of motion during the Z- and A-modes trials (cyan and red bars, respectively) are shown for amputee groups (mean values ± SD). The time axes in panels **A** and **B** start at the heel strike of the unperturbed gait cycle (grey/shadow area) and at the onset of the perturbed strides (blue and red lines).

The hip range of motion (ROM) of the PL significantly changed among conditions (i.e., no-APO, Z-mode and A-mode; one way repeated measures ANOVA, N = 8, p = 0.001 for elderly subjects; Fig. 3C). The post-hoc Tukey test revealed that the hip ROM of the PL was significantly different during the A-mode trials compared to the other two conditions (p = 0.002 and p = 0.039, respectively for no-APO and Z-mode). Specifically, values related to A-mode were lower than the other two conditions (Z-mode and no-APO), which instead appeared comparable (Fig. 3C).

Conversely, the hip ROM of the UL was not modified among conditions (i.e., no-APO, Z-mode and A-mode; one way repeated measures ANOVA, N = 8, p = 0.635 for elderly subjects; Fig. 3C).

Concerning the amputees, the behavior of these participants was comparable to that observed for elderly people. Specifically, the hip ROM of the PL, for amputees, was lower when APO was set in A-mode than during Z-mode (Fig. 3D). Differently, no modifications were observed at hip ROM of the UL (Fig. 3D).

### Stability against balance loss: effects of the proposed approach

To investigate whether our approach was effective in promoting balance recovery, the COM stability^19^,^20^,^21^ and the margin of stability (MOS)^22^,^23^ were computed. These metrics were calculated at the touch down of the perturbed leg (TD_P_) to ensure that the assistive action, when present, was completed. Additional details are reported in Materials and Methods, Section E.

**Figure 4:**
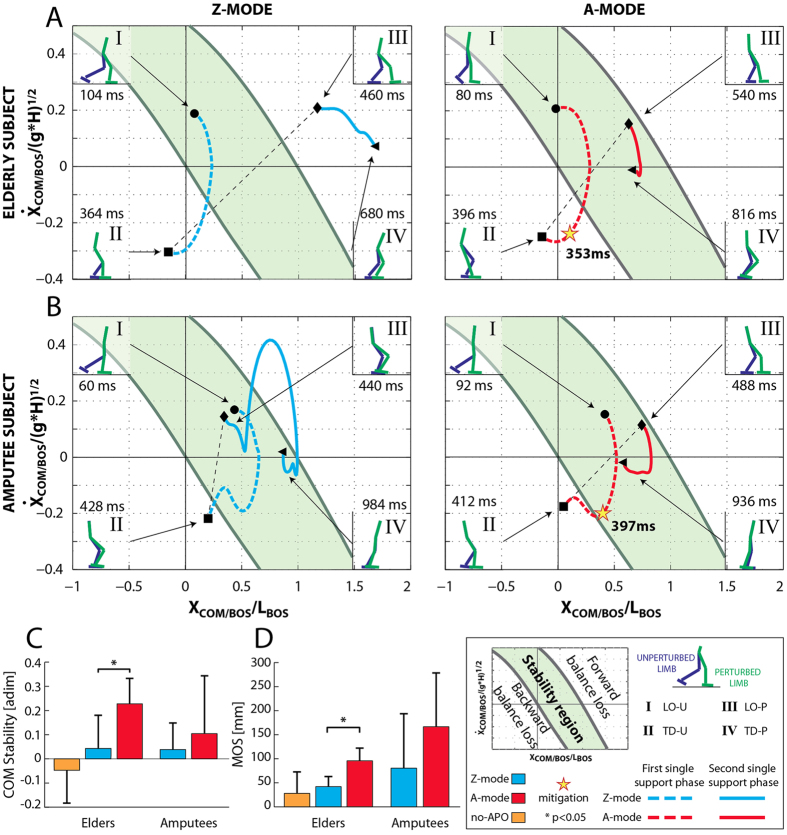
Analysis of the stability against fall. Subplots (**A** and **B**) show the COM motion state for one elderly subject and one amputee, during the Z- and A-modes (cyan and red lines, respectively). The green area represents the stability region: forward falls would be initiated if states exceeded the upper boundary; backward falls would be initiated if states dropped below the lower boundary. The two components of the COM motion state (i.e., position and velocity of the COM) were calculated relative to the base of support (BOS) and normalized by foot length and (g*H)^1/2^, respectively, where g is gravitational acceleration and H is body height. Two single support phases, concerning the PL and UL, were identified from the lift-off and the touch-down of the unperturbed (I-II events, LO_U_ and TD_U_) and the perturbed (III-IV events, LO_P_ and TD_P_) feet, respectively. The effect of the fall mitigation action was observed during the second single support phase, and, accordingly, the dynamic stability was assessed at the end of the compensatory stride (i.e., IV event). (**C**) COM stability and (**D**) margin of stability (MOS) are depicted for the no-APO, Z- and A-modes (orange, cyan and red bars respectively), for both elderly and amputee groups (mean values ± SD). The label * indicates a significant (p < 0.05) difference among trials.

The time course of the COM motion state is shown in a representative case for both elderly subjects and amputees wearing the APO (Fig. 4A and B, respectively). During the first single support phase (I–II events, lift off to touch down of the unperturbed leg, LO_U_ -TD_U_, red/cyan dashed lines), intra-subject differences were not observed between the Z- and A- modes because the mitigation strategy, when it was enabled, was not yet activated.

During the second single support phase (III-IV events, lift off to touch down of the perturbed leg, LO_P_–TD_P_, red/cyan solid lines), we observed different behaviors between Z-mode and A-mode for both the healthy subject and the amputee. Specifically, when the APO was functioning in Z-mode, the COM motion state of the healthy subject moved outside the stability region and ended above the limit of the forward balance loss region; whereas the COM motion state of the amputee had a long transition to the instability region and ended close to its limit. By contrast, when the APO was functioning in A-mode, and the mitigation strategy was enabled (see yellow stars in Fig. 4A and B), the COM motion state was confined within the stability region for all the duration of the second single support phase (III–IV events, LO_P_ –TD_P_, red/cyan solid lines), thus facilitating the balance recovery of both healthy and amputee subjects (see Supplementary Movies S1 and S2).

The one-way ANOVA on COM stability and MOS yielded significant variation among the three conditions in elderly subjects (i.e., no-APO, Z-mode and A-mode; N = 8, p = 0.019 and p = 0.011 respectively; Fig. 4C and D, respectively). In this respect, the post hoc Tukey test revealed COM stability (p = 0.040) and MOS (p = 0.047) were significantly higher during A-mode condition than during the Z one, thus highlighting an improvement in overall balance against falls due to the assistive strategy of the exoskeleton.

Noticeably, the adopted mitigation strategy promoted balance recovery in transfemoral amputees, as well. As a matter of fact, both metrics (i.e., COM stability and MOS) were higher during A-mode than Z-mode (Fig. 4C and D).

### Analysis of the obtrusiveness of the APO

During unperturbed locomotion (i.e., before the onset of the perturbation), the kinematic patterns of the elderly subjects and amputees, while wearing the APO, were similar to those reported in literature (Fig. 3A and B, respectively, grey curves^24^,^25^). Thus, the exoskeleton appeared likely transparent while participants walked steadily, confirming previous results^26^.

As far as the compensatory stride after the perturbation is concerned, the pairwise Tukey’s comparison revealed that, during no-APO trials the ROM at both hip joints (i.e., PL and UL) was not significantly different (p = 0.080 and p = 0.640, respectively) than that observed when the APO was set in Z-mode (Fig. 3C). In addition, the statistical analysis also revealed that both COM stability and MOS observed during the Z-mode were not significantly different than that seen when subjects were not wearing the exoskeleton (i.e., no-APO condition; p = 0.702 and p = 0.409, respectively; Fig. 4C and D, respectively).

According to these evidences, the reactive behavior of elderly participants was not altered by the use of the exoskeleton in Z-mode in terms of both kinematics and stability, thus revealing that the APO was unobtrusive with respect to the experimental conditions.

## Discussion

All these findings indicate that the light-weighted APO can detect in real-time the onset of a mechanical perturbation and activate specific mitigation strategies to promote balance recovery. Specifically, our experimental design revealed that the proposed strategy effectively (p < 0.05) favorites the balance recovery in elderly people after a slippage. Moreover, our pilot tests on transfemoral amputees showed that these subjects did benefit from the APO assistance in the same perturbing conditions. We acknowledge that further experimental sessions are required to robustly confirm these latter results. However, these findings suggest that the proposed approach is easily adaptable to different groups of prone to fall subjects, and represents a valuable proof of concept concerning the attitude of wearable robotic devices to assist persons while managing challenging motor tasks.

Remarkably, our detection algorithm identified the lack of balance in about 350 ms (Fig. 2B), consistent with the performance estimated during our previous off-line study^16^ and compatible with a possible falling avoidance^27^,^28^. In addition, our approach does not require any subject-specific and training-based customizing procedure for successful implementation among different subjects. In fact, the algorithm for perturbation detection is based on the comparison between the actual leg joint angles and those predicted by a pool of adaptive oscillators (Fig. 2A). Once the algorithm’s tuning was optimized^16^, it was immediately able to detect postural transitions without any further subject-based training. Moreover, the amplitude of the torque was easily tuned taking into account only the body weight of the subjects (see Materials and Methods, Section B).

Another very interesting characteristic of our approach is that it promotes users’ balance recovery only if required by providing an additional “impulsive” torque achieving the ecological and symbiotic approach previously mentioned. Accordingly, our “assisting when needed” strategy suggests the development of a novel concept of wearable devices specifically designed to minimize the risk of falling. In this respect, we envisage that a more focused design would further reduce the bulkiness of wearable robotic platforms and increase usability and acceptance by senior and disabled users. This opportunity requires further investigations to identify the APO with the minimum performance necessary to effectively reduce fall risk.

After this promising proof of concept, challenges lie ahead. Our results must be confirmed in additional subjects and with different types of mechanical perturbations. It will be extremely important to understand the limits of both the detection and the mitigation algorithms under different experimental conditions and, subsequently, in real-life field tests. Indeed, a loss of balance can be induced by a number of causes, such as multi-directional slipping-like perturbations, tripping, and stumbling. In addition, the psycho-physical status of fragile persons further increases the risk of falls.

Overall, our findings demonstrate the potentials of ecologically-controlled wearable robots to assist the elderly and disabled subjects during slipping events, thus potentially improving their quality of life.

## Methods

### Participants and experimental setup

Ten subjects, 2 transfemoral amputees and 8 age- and anthropometry-matched elderly subjects (see Table S1) were enrolled for this study. The exclusion criteria were: age greater than 75 years old, falls documented in the last 6 months, relevant comorbidities (e.g., hemiplegia, degenerative nervous system diseases, chronic heart failure, chronic obstructive pulmonary disease, sever sensory deficits), poor cognitive skills (Mini-Mental State Examination score <24), inability to walk safely on a treadmill, and severe anxiety or depression. In addition, eligible amputees had, at a minimum, the ability to transfer using a household ambulator on a level surface (Medicare Functional Classification Levels K-level ≥K2). Enrolled subjects were informed about the purpose of the study and signed informed consent forms before the experimental sessions began. All research procedures were in accordance with the Declaration of Helsinki and were approved by the Institutional Ethics Committee of Don Gnocchi Foundation (Florence, Italy), where the experiments took place.

Subjects were asked to manage unexpected slipping-like perturbations delivered by a custom-made split-belt treadmill which belts can be independently controlled to provide multi-directional perturbations^18^, (Fig. 1 and Fig. S2, Supplementary Materials and Methods, Section A). Subjects donned a safety harness attached to an overhead track during the trials for safety-related issues.

The perturbation consisted of a forward movement of one belt at the heel strike of the foot being perturbed^29^,^30^. Two representative velocity profiles of the belts related to the perturbed and the unperturbed limbs are presented in Fig. S2. Perturbations were delivered on the prosthetic limb and on the right side for amputees and elderly subjects, respectively. Amputees donned their own standard prostheses and all participants used their own athletic shoes during the entire experimental session. Noticeably, the perturbation was delivered on the prosthetic side in order to allow the amputees to safety rely on the sound limb during the compensatory step. Otherwise, if the perturbation was delivered on the sound limb, the amputees would suddenly transfer their body weight from the sound limb to the prosthesis. In this condition, the prosthetic knee would be unlocked (i.e., it would be supposed to start a swing phase) thus collapsing under the amputee’s weight and leading to a fall.

During the experimental sessions, participants wore a novel APO, a powered wearable device^17^, designed to assist hip flexion-extension in the sagittal plane (Fig. 1 and Fig. S1; more details in Supplementary Materials and Methods, Section B). The control strategy driving the APO can switch between two working modalities, i.e., zero-torque and assistive modes (Z- and A-modes, respectively). The former (Z-mode) is designed to allow the wearer to freely perform movements without being obstructed by the device. The latter (A-mode) is enabled only when a balance loss is detected and supplies torques at both hip joints to promote stability recovery (more details in Materials and Methods, Section B).

The postural transition was detected by a plug-and-play algorithm based on adaptive oscillators and running in the APO’s control unit (Supplementary Materials and Methods, Section C)^16^. Once the perturbation was detected, the exoskeleton switched from Z-mode to A-mode, and the APO supplied the counteracting torques. The assistive strategy (A-mode) consisted in increasing the stiffness at hip joints against limb movements induced by the belts, thus counteracting the downward movement of the body COM. In this regard, when the A-mode was enabled, torque patterns acted in order to extend the perturbed limb and flex the unperturbed one (more details in Materials and Methods, Section B).

Furthermore, a subset of five elderly subjects performed additional trials without wearing the APO in order to test the hypothesis that the APO was unobtrusiveness with respect to the experimental conditions.

### Assistive strategy

Literature revealed that after a slip, reactive joint moments at the knee and hip joints are mostly aimed at slowing down the sliding motion of the foot and, possibly, at minimizing the downward displacement of the body. In particular, the reactive moment at the hips is responsible for the lowering of the foot onto the ground, while the reactive moment at the knees decelerates the trailing leg and absorbs part of the energy generated by the hip extensors^31^.

According to these evidences, we hypothesized that an increment of the stiffness at hip joints against limb movements induced by the belts could promote the balance recovery. In this regard, when the A-mode is enabled, torque patterns acted in order to extend the perturbed limb and flex the unperturbed one. The assistive strategy implemented in the APO was defined as a time-dependent torque pattern which profile was designed based on two settable features: *A* [Nm], the amplitude; *T* [ms], the duration. Specifically, if the perturbation is detected at t^*^, the set point of the counteracting torques was defined as follows for t* ≤ *t* ≤ t* + *T* (elsewhere *τ*_*des*_ was zero; see Supplementary Methods, Section B online):


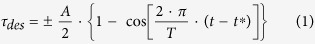


where ± refers to flexor and extensor torques, respectively.

Despite the implemented strategy was defined in accordance with physiological countermeasure described in literature^31^, the duration and the intensity of the assistive torque are two critical issues which have never been investigated before. As a matter of fact, from the best of our knowledge, no authors have ever closed the loop from detecting an incipient fall to provide a suitable support by means of a wearable robotic platform. Therefore, to address these issues, we adopted following assumptions:

(i). Since a fall occurs in 0.7–1 s^29^ and the detection time is expected to be about 0.3–0.4 s^12^, the duration of the assistive torque was set at 0.25 s.

(ii). Since the reactive response depends on the subject inertia, the APO provided a torque proportional to the gross (participant + exoskeleton) weight, that is, 0.2 Nm/kg.

### Data collection

The protocol consisted of two repetitions for each of the following experimental conditions: assisted (i.e., A-modes); no-assisted (i.e., Z-mode); without wearing the exoskeleton (i.e., no-APO) trials. To reduce the effect of the adaptation on the results: i) the full experimental session accounted for three additional trials, in which no perturbation was delivered; ii) all (perturbed and no-perturbed) trials were randomly arranged; iii) participants did not know whether they would be perturbed or not; and iv) participants did not know whether they would be assisted or not.

The 3D kinematics of a set of spherical markers located on suitable body landmarks of the whole body and on the APO were recorded at 250 Hz using a six-camera-based Vicon 512 Bonita 10 Motion Analysis System (Oxford, U.K.) (more details in Supplementary Materials and Methods, Section D).

Body kinematics records, belt movements and the APO were synchronized using a logic pulse generated by the split-belt treadmill during the delivery of the perturbations.

### Data processing

Raw data were firstly pre-processed to remove the effects of noise and artifacts. In particular, high-frequency related noise was removed from digitized coordinates by low-pass filtering data (zero-lag, 4^th^ order Butterworth low-pass filter) with cut off at 10 Hz. The cut off frequency was selected as described elsewhere^32^. Raw ground reaction force signals were also band-pass-filtered (Butterworth filter, 4^th^ order, cutoff at 0.5–10 Hz) in accordance with previous authors^33^,^34^.

A full body model accounting for 15 segments and 42 internal degrees of freedom was developed. The 15 segments were: head/neck, chest, abdomen/pelvis, upper arms, forearms, hands, thighs, shanks and feet. All joints were approximated as spherical and their center was located in accordance with the literature^30^,^35^,^36^,^37^,^38^. For the *i*^*th*^ body segment, an orthogonal local reference frame was located in its own center of mass and defined according to ISB recommendations^39^. The time course of limb joints angles into the sagittal plane were estimated by using the 3D approach described in literature^39^. The range of motion (ROM) at hip joints was computed as the difference between the maximum and the minimum values during the compensatory cycle (i.e., from the onset of the perturbation to the touch down of perturbed leg, TD_P_).

Body segment inertial parameters (i.e., mass and center of mass position) were calculated using procedures described by Zatsiorsky and colleagues^40^, and modified by de Leva^41^. The inertial properties of the prosthesis were considered equal to those of the sound limb. Mass and center of mass of each of the body segments were used to estimate the whole body center of mass (COM).

Touch down and lift off were identified by visual inspection of kinematics of the feet and time course of the vertical component of the ground reaction force.

For each subject and trial, data were subdivided in two subsets: data recorded before and after the onset of the perturbation. The former referred to the last three ipsilateral unperturbed strides, in which each cycle started and finished with the heel strike of the leg being perturbed. These data were subdivided into strides, individually time-interpolated over 250 points, and averaged in order to have a representative unperturbed gait cycle. After the touch down of the foot being perturbed, four consecutive time events were identified: (I) the lift off of the unperturbed foot (LO_U_); (II) the instant immediately before (i.e., 1 frame, consisting of 1/250 s) the touch down of the unperturbed foot (TD_U_); (III) the lift off of the perturbed foot (LO_P_); and (IV) the instant immediately before the touch down of the perturbed foot (TD_P_). Overall, these four consecutive time events allowed for investigating stability against balance loss, under the hypothesis that the balance recovery may require more than one step to be achieved.

### Analysis of the stability against balance loss

To investigate the effectiveness of the assistive strategies while promoting balance recovery, the following metrics were calculated: margin of stability (MOS) and COM stability.

The MOS along the antero-posterior (AP) direction was computed according to Hof *et al*.^23^ as:


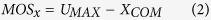


where U_MAX_ is the anterior boundary of the base of support (BOS) and X_COM_ is the position of the extrapolated COM in the AP direction estimated as follows:


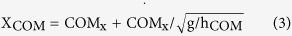


where COM_x_ and 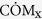 are the AP components of position and velocity of the COM, h_COM_ is the distance between COM and floor while subjects were keeping the unperturbed upright stance, and g is the gravitational acceleration. The MOS was quantified at the end of the compensatory stride (i.e., TD_P_).

The COM motion state was used to investigate the ongoing of the balance recovery. This variable has been introduced by previous authors^19^,^20^,^21^, and indicates the range of values for both COM position and velocity to allow a feasible control of subject’s stability. Specifically, based on these state variables it is possible to determine upper and lower boundaries identifying a feasible stability region against backward or forward balance loss. The COM stability was hence defined as the shortest distance from the instantaneous COM motion state to the limits against backward or forward balance loss^21^ and was calculated at TD_P_.

### Statistical analysis

Mean and standard deviation were the main descriptive statistics used to summarize the main features of data samples. With respect to elderly subjects, the effect of the exoskeleton (i.e., Z-mode vs. A-mode vs. no-APO) on hip ROM, COM stability and MOS was assessed by means of one-way repeated measures ANalysis Of VAriance (ANOVA). If significant, this analysis was followed up with Tukey Honestly Significant Difference test in order to test two different hypothesis: i) the proposed assistive strategy (i.e., A-mode) favorites the balance recovery compared to the Z-mode; ii) the reactive response of the subjects is not altered when the exoskeleton works in Z-mode, i.e., the device is not obtrusive with respect to the experimental conditions.

Data processing was performed using custom written MATLAB (The MathWorks, Inc., Natick, MA, USA) scripts; the statistical analysis was carried out by means of Minitab (Minitab Inc., PA, USA). Significance of statistical tests was set at α = 0.05.

## Additional Information

**How to cite this article:** Monaco, V. *et al*. An ecologically-controlled exoskeleton can improve balance recovery after slippage. *Sci. Rep.* **7**, 46721; doi: 10.1038/srep46721 (2017).

**Publisher's note:** Springer Nature remains neutral with regard to jurisdictional claims in published maps and institutional affiliations.

## Supplementary Material

Supplementary Information

Supplementary Video 1

Supplementary Video 2
