# Supplementary material for: An ecologically-controlled exoskeleton can improve balance recovery after slippage
Source: Sci Rep. 2017 May 11;7:46721. doi: 10.1038/srep46721 (PMC5426188; doi:10.1038/srep46721)
Supplement: Supplementary Information [file srep46721-s1.doc]

An ecologically-controlled exoskeleton can improve balance recovery after slippage

**Authors:** V. Monaco^1,2*^, P. Tropea^1^, F. Aprigliano^1‡^, D. Martelli^1,3‡^, A. Parri^1^, M. Cortese^1^, R. Molino-Lova^2^, N. Vitiello^1,2†^, S. Micera^1,4*†^

**Affiliations:**

^1^ The BioRobotics Institute, Scuola Superiore Sant’Anna, Pisa, Italy

^2^ IRCSS Don Carlo Gnocchi Foundation, Firenze, Italy

^3^ Department of Mechanical Engineering, Columbia University, New York, NY 10027 USA

^4^ Bertarelli Foundation Chair in Translational NeuroEngineering, Center for Neuroprosthetics and Institute of Bioengineering, School of Engineering, Ecole PolytechniqueFederale de Lausanne, Lausanne, Switzerland

*To whom correspondence should be addressed: [silvestro.micera@santannapisa.it[epfl.ch](mailto:silvestro.micera@santannapisa.it%5Bepfl.ch)], [vito.monaco@santannapisa.it](mailto:vito.monaco@santannapisa.it)

† Equal contributors as senior authors.

‡ Equal contributors as junior authors.

# List of abbreviations

A-mode Assistive modality

APO Active Pelvis Orthosis

COM Center Of Mass

LO_P_ Lift Off of the Perturbed foot

LO_U_ Lift Off of the Unperturbed foot

MOS Margin Of Stability

PL Perturbed Limbs

ROM Range Of Motion

TD_P_ Touch Down of the Perturbed foot

TD_U_ Touch Down of the Unperturbed foot

UL Unperturbed Limbs

Z-mode Zero-torque modality

# Supplementary Figure S1


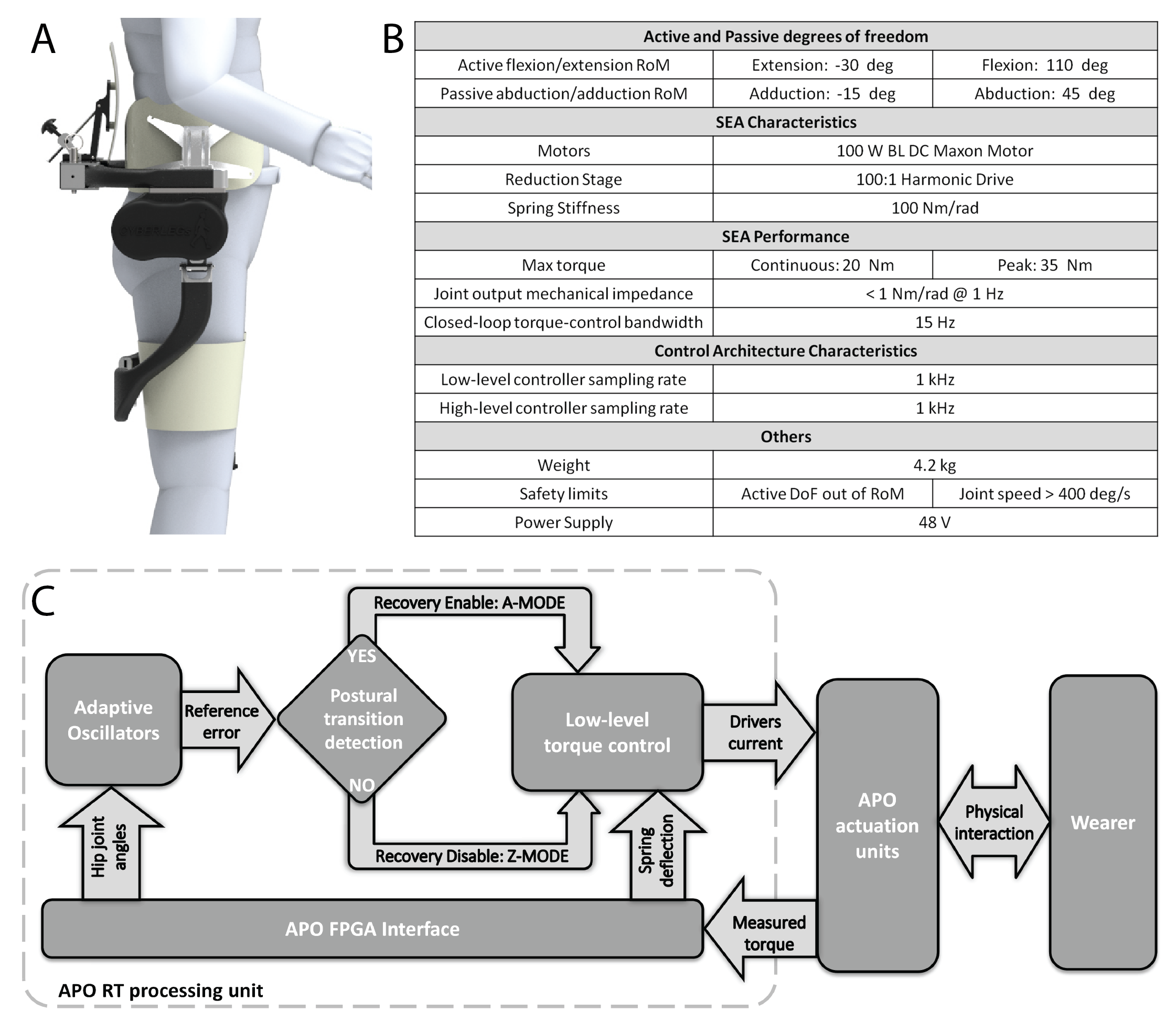


# Supplementary Figure S1. Active Pelvis Orthosis. (A) Lateral view of the APO. (B) Summary of main characteristics and performance of the APO mechatronic architecture. (C) Conceptual block diagram of the control architecture. The hip joint angles measured from the real time processing unit are the input of the adaptive oscillators block which continuously updates the reference trajectory thanks to its inherent learning features. The postural transition detection consists of the threshold based algorithm described in Fig. 2: if the postural transition is detected, A-mode is enabled and the set point of the low-level control loop is the counteracting torque, otherwise, the set point is forced at zero. In the latter case (Z-mode) the APO follows the user's movement without hindrance. Panel A was created by Mr. Francesco Giovacchini.

# Supplementary Figure S2

**
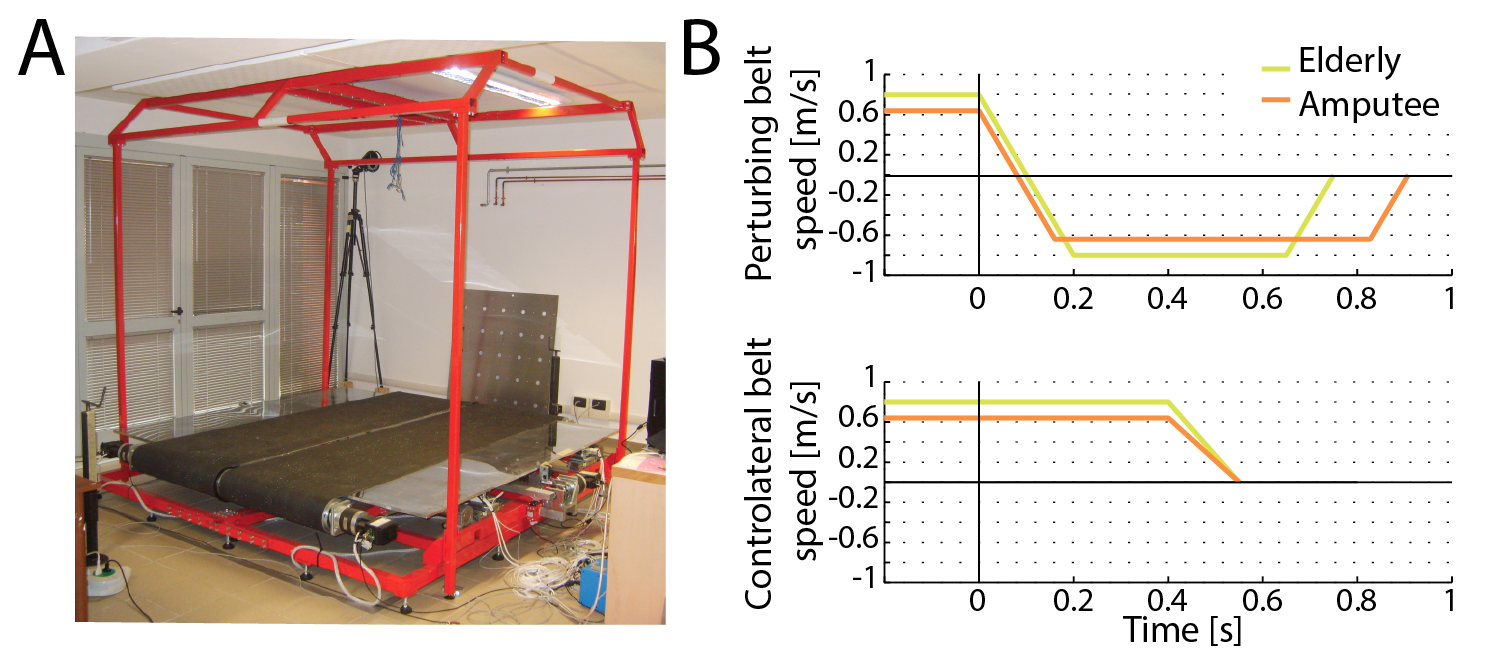
**

# Supplementary Figure S2. Mechatronic split-belt treadmill (A) Mechatronic platform designed to deliver multi-directional slipping-like perturbations. (B) Velocity profiles of belts related to the perturbed (top) and contralateral (bottom) limbs for an older adult and an amputee (yellow and orange, respectively). The origin of the time frame is set at the onset of the perturbation.

# Supplementary Table S1

**Supplementary Table S1. Anthropometric features and walking speed for all subjects belonging to the elderly group (EG) and amputee group (AG).**

| Group | Subject ID | Age [years] | Weight [kg] | Height [m] | Leg Length [m] | Speed [m/s] | Gender |
| --- | --- | --- | --- | --- | --- | --- | --- |
| EG | E01 | 74 | 66 | 1.69 | 0,78 | 0,91 | Male |
|  | E02 | 65 | 85 | 1.80 | 0,83 | 0,99 | Male |
|  | E03 | 69 | 71 | 1.82 | 0,86 | 0,90 | Male |
|  | E04 | 68 | 70 | 1.77 | 0,84 | 1,00 | Male |
|  | E05 | 76 | 79 | 1.78 | 0,83 | 0,70 | Male |
|  | E06 | 73 | 70 | 1.65 | 0,73 | 0,85 | Male |
|  | E07 | 63 | 90 | 1.80 | 0,86 | 0,80 | Male |
|  | E08 | 62 | 81 | 1730 | 0,81 | 1,00 | Male |
|  | **Mean** | **68.9** | **76.5** | **1.75** | **0.82** | **0.89** |  |
|  | **St. dev.** | **5.06** | **8.50** | **0.59** | **0.04** | **0.11** |  |
| AG | A01 | 61 | 68 | 1.80 | 0.84 | 0.64 | Male |
|  | A02 | 70 | 80 | 1.70 | 0.79 | 0.73 | Male |
|  | **Mean** | **65.5** | **74** | **1.75** | **0.82** | **0.69** |  |
|  | **St. dev.** | **6.36** | **8.49** | **0.07** | **0.04** | **0.06** |  |

# Supplementary Methods

## Mechatronic split-belt treadmill

SENLY [^1^](#_ENREF_1) is a custom-made mechatronic platform mainly consisting of a two split-belt treadmill (see Fig. S2). The belts can be independently moved both longitudinally and transversally (i.e., the right belt can be moved in all clockwise directions from north to south, and the left belt can be moved in all directions in the remaining hemiplane) and are wrapped around two platforms provided with force cells. It allows for supplying slipping-like perturbations when a particular distribution of load between the two platforms is detected, thus to control the onset of the perturbation across experimental sessions. Participants donned a safety harness, successively attached to an overhead track in order to prevent impacts with the ground in case of falling but without restricting their movements. The global reference frame was located at the centre of platform with X axis along the anterior/posterior direction, Y axis vertical and Z axis defined by the right-hand rule along the medial/lateral direction.

The perturbation consisted of a trapezoidal speed profile of the perturbing belt which amplitude was normalized with respect the subject’s walking speed and leg length (see Fig. S2). More in detail, when the perturbation was enabled, the belt was accelerated (8 m/s^2^) in the opposite direction until its speed reached the same value set during steady walking. Then, its velocity was kept at fixed value and finally brought to zero with deceleration of 8 m/s^2^, in order to cover a total treadmill displacement equals to 60% of the leg length. The speed of the contralateral belt was maintained at the walking speed for 0.4 s and then brought to zero in 0.15 s with 8 m/s^2^ of deceleration.

## Active Pelvis Orthosis (APO)

The APO [^2^](#_ENREF_2) is conceived as a cooperative robotic technology designed to assist hip flexion/extension movements of people with mild locomotion impairments during walking. Specifically, the APO is constituted of a C-shaped carbon fibre frame enveloping the trunk and of two carbon fibre links, coupled to the actuation units, interfaced distally with wearer’s thighs (see Fig. S1). A comfortable physical human-robot interface is ensured by five orthotic shells coupled with user trunk and thighs.

An active degree of freedom (DoF) powers the hip flexion-extension with a range of motion of [-20 ÷ 110] deg. Furthermore, linkages endowed a passive DoF for abduction-adduction [-15 ÷ 25] deg. Two series elastic actuators (SEAs) [^3^](#_ENREF_3), endowing a patented torsional spring [^4^](#_ENREF_4), provide compliant and safe actuation with minimum joint output impedance across the frequency spectrum of gait (≈ 1 Nm/rad @ 1 Hz) [^2^](#_ENREF_2). The choice of SEAs was dictated by the fact they have been successfully applied in the field of wearable powered robots mostly to solve safety issues and reduce the inherent output impedance of the actuated joint [^5-7^](#_ENREF_5). The APO is also provided with several additional and passive DoFs which allow for adjusting the position of actuated rotational axes and the dimensions of the frame in order to fit a wide range of anthropometries.

The control system runs at 1 kHz on a real time controller, a cRIO-9082 (National Instruments, Austin, Texas, US), endowed with both a 1.33 GHz dual-core processor running a NI real time operating system, and a field programmable gate array (FPGA) processor Spartan-6 LX150. The APO is controlled by means of a hierarchical architecture: i) a low-level layer is responsible for driving the SEA units by means of a closed-loop torque controller; it relies on a PID regulator operating on the error between the set point and the measured torque (being known the deflection of the torsional spring); ii) a high-level control layer implements adaptive assistive strategies for locomotion assistance and can switch between two operative conditions according to the time course of the observed state variables. In particular, during the zero-torque mode, namely Z-mode, the APO controller is asked to continuously delete the displacement of the torsional spring induced by human volitional movements, thus resulting extremely compliant to the user’s intention. This behaviour is achieved by setting the torque reference of the low-level torque controller as
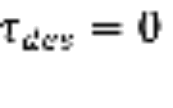
 *Nm*. In this study, the assistive mode, namely A-mode is enabled only when a balance loss is detected and consists in supplying torques at both hip joints to promote stability recovery. In both cases (Z- and A-mode), PID gains were tuned in order to achieve a maximum closed-loop bandwidth of about 15 Hz.

## Real time detection of slipping-like perturbations

The lack of balance was detected in real time by a threshold-based algorithm comparing the actual kinematics of the robot with that predicted by a pool of adaptive oscillators (AOs) [^8^](#_ENREF_8). This approach grounds on the hypothesis that the quasi-periodic features of the kinematics of steady walking are abruptly modified after a postural transition. Therefore, the discrepancy between the actual kinematics and that predicted by a pool of AOs tuned to correctly track the steady walking can allow for an effective detection of the onset of the postural transition. Hereafter, its working principle is reported.

More in detail, an AO is a mathematical predictor able to synchronize its output to a quasi-periodic input signal while learning its relevant features, i.e., phase, frequency and amplitude [^9^](#_ENREF_9). As result, the signal estimated by the AO is, on average, delay-free with respect to the actual input signal [^10^](#_ENREF_10). When the dynamics of the AO is sufficiently slow, an abrupt modification of the time course of the observed variables cannot be properly tracked, thus involving a significant deviation between desired and estimated signals. Accordingly, the AO can be easily used to monitor quasi-periodic kinematic patterns during steadily walking and highlight their quick modifications resulting after unexpected balance loss.

With respect to the purpose of the study, the onset of the postural transitions induced by the mechatronic platform was detected by analysing the difference between actual (i.e., ) and estimated (i.e.,) hip joint angles (i.e.,
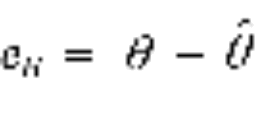
). Specifically, for each time series
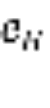
 our threshold-based algorithm achieved the following steps:

1. the mean (μ) and the standard deviation (σ) along a *m*-sample long time window were computed;
2. the thresholds were set as
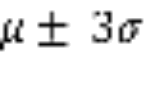
;
3. a warning was recorded if the first sample of the observed time window was over the threshold; otherwise, the warning stack was reset;
4. the time window moved forward by one sample and the algorithm started again from the step *a)*;
5. when *r* consecutive warnings were collected, the algorithm stopped and the postural transition was detected.

The whole domain of the AOs dynamics (i.e., the full range of phase and amplitude gains) at different setting conditions (i.e., *m* and *r*) was analysed in a previous work [^8^](#_ENREF_8) identifying the optimal tuning of the algorithm in order to easily detect an unexpected loss of balance due to a slippage. In particular, optimal performance were obtained by minimizing mean detection time (i.e., the time elapsing between the onset of the perturbation and the output of the algorithm) and false alarms.

## Marker set

During the experimental sessions the 3D trajectory of 34 markers located on suitable body landmarks and 10 on the APO was recorded. Bilateral body landmarks were the following: vertex, left and right gonions, for the head; C7 vertebrae, clavicle, sternum, and acromions, for the upper trunk; lateral epicondyle of the homers, radial styloids, ulnar styloids, third metacarpal bones and additional markers rigidly attached to wands over the mid-homers, for both arms; lateral epicondyle of the femurs, heads of fibula, lateral malleolus, calcaneus, first and fifth metatarsal heads, and additional markers rigidly attached to a wand over the mid-femurs and mid-shaft of the tibia, for both legs. Ten markers were attached to the APO, four on the pelvis pad and three on each of the pad wrapping thighs.

Before the trials, a static calibration procedure was carried out for every subject placing 7 additional markers on: T10 vertebrae, for the upper body; medial epicondyle of the homers, for both arms; medial epicondyle of the femurs and medial malleolus, for both legs. In addition, the location of further 5 landmarks (i.e., the anterior superior iliac spines, the sacrum and the prominence of the greater trochanters external surface) was also recorded using a pointer since they were covered by the APO.

## References

1 Bassi Luciani, L. *et al.* Design and evaluation of a new mechatronic platform for assessment and prevention of fall risks. *J Neuroeng Rehabil* **9**, 51 (2012).

2 Giovacchini, F. *et al.* A light-weight active orthosis for hip movement assistance. *Robotics and Autonomous Systems* **73**, 123-134, doi:http://dx.doi.org/10.1016/j.robot.2014.08.015 (2015).

3 Pratt, G. A. & Williamson, M. M. in *Intelligent Robots and Systems 95. 'Human Robot Interaction and Cooperative Robots', Proceedings. 1995 IEEE/RSJ International Conference on.* 399-406 vol.391.

4 Giovacchini, F., Cempini, M., Vitiello, N. & Carrozza, M. C. Molla Torsionale. Italy patent (2013).

5 Veneman, J. F. *et al.* Design and Evaluation of the LOPES Exoskeleton Robot for Interactive Gait Rehabilitation. *Neural Systems and Rehabilitation Engineering, IEEE Transactions on* **15**, 379-386, doi:10.1109/TNSRE.2007.903919 (2007).

6 Vitiello, N. *et al.* NEUROExos: A Powered Elbow Exoskeleton for Physical Rehabilitation. *Robotics, IEEE Transactions on* **29**, 220-235, doi:10.1109/TRO.2012.2211492 (2013).

7 Zinn, M., Khatib, O., Roth, B. & Salisbury, J. K. in *Experimental Robotics VIII* Vol. 5 *Springer Tracts in Advanced Robotics* (eds Bruno Siciliano & Paolo Dario) Ch. 9, 113-122 (Springer Berlin Heidelberg, 2003).

8 Tropea, P. *et al.* Detecting Slipping-Like Perturbations by Using Adaptive Oscillators. *Annals of biomedical engineering*, doi:10.1007/s10439-014-1175-5 (2014).

9 Righetti, L. & Ijspeert, A. J. in *Robotics and Automation, 2006. ICRA 2006. Proceedings 2006 IEEE International Conference on.* 1585-1590.

10 Ronsse, R. *et al.* Oscillator-based assistance of cyclical movements: model-based and model-free approaches. *Med Biol Eng Comput* **49**, 1173-1185, doi:10.1007/s11517-011-0816-1 (2011).

# Supplementary Movie S1

**Elderly subject.** The participant donned the APO and walked steadily on the split-belt treadmill until the perturbation was delivered. The left and the right panels show the subject’s biomechanical response when the APO functioned under Z-mode and A-mode, respectively. Specifically: during the Z-mode no assistance was provided to the user; during the A-mode, the APO supplied counteracting torques at hip joints and assisted the subject in balance recovery.

# Supplementary Movie S2

**Trans-femoral amputee.** The participant donned the APO and walked steadily on the mechatronic platform until the perturbation was delivered. The left and the right panels show the amputee’s biomechanical response when the APO functioned under Z-mode and A-mode, respectively. Specifically: during the Z-mode no assistance was provided to the user; during the A-mode, the APO supplied counteracting torques at hip joints and assisted the amputee in balance recovery.
